# A coordination polymer of Cd^II^ with benzene-1,3-dicarboxyl­ate and 1,4-bis­[1-(2-pyridylmeth­yl)benzimidazol-2-yl]butane

**DOI:** 10.1107/S1600536809043360

**Published:** 2009-10-28

**Authors:** Wei-Ping Zhang, Wen-Xiu Zhao, Gui-Hua Cui, Shun-Fu Dong

**Affiliations:** a Jilin Medical College, Jilin 132013, People’s Republic of China

## Abstract

The title Cd^II^ coordination polymer, *catena*-poly[[{1,4-bis­[1-(2-pyridylmeth­yl)benzimidazol-2-yl]butane}cadmium(II)]-μ-benzene-1,3-dicarboxyl­ato], [Cd(C_8_H_4_O_4_)(C_30_H_28_N_6_)]_*n*_, was obtained by reaction of CdCO_3_, benzene-1,3-dicarboxylic acid (H_2_btc) and 1,4-bis­[1-(2-pyridylmeth­yl)benzimidazol-2-yl]butane (*L*). The Cd^II^ cation is six-coordinated by an N_2_O_4_-donor set. *L* acts as a bidentate ligand and btc anions link Cd^II^ centers into a chain propagating parallel to [010].

## Related literature

For the potential applications of metal-organic coordination polymers, see: Zhao *et al.* (2008 ▶). For related structures, see: Liu *et al.* (2007 ▶); Zhang *et al.* (2008 ▶).
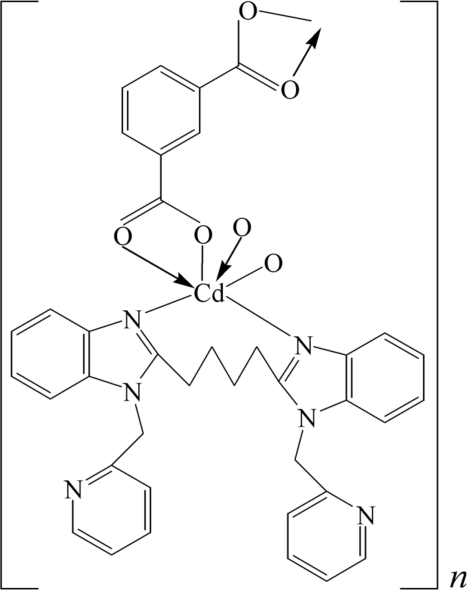


## Experimental

### 

#### Crystal data

[Cd(C_8_H_4_O_4_)(C_30_H_28_N_6_)]
*M* *_r_* = 749.10
Triclinic, 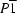

*a* = 8.999 (4) Å
*b* = 10.094 (5) Å
*c* = 19.135 (9) Å
α = 91.569 (2)°
β = 97.412 (2)°
γ = 107.297 (1)°
*V* = 1641.7 (13) Å^3^
*Z* = 2
Mo *K*α radiation
μ = 0.72 mm^−1^
*T* = 293 K
0.21 × 0.18 × 0.10 mm

#### Data collection

Bruker APEX CCD area-detector diffractometer
Absorption correction: multi-scan (*SADABS*; Sheldrick, 1996 ▶) *T*_min_ = 0.85, *T*_max_ = 0.92
16049 measured reflections
7395 independent reflections
5023 reflections with *I* > 2σ(*I*)
*R*_int_ = 0.054

#### Refinement

*R*[*F* ^2^ > 2σ(*F* ^2^)] = 0.052
*wR*(*F* ^2^) = 0.135
*S* = 1.09
7395 reflections
442 parameters
H-atom parameters constrained
Δρ_max_ = 0.62 e Å^−3^
Δρ_min_ = −0.73 e Å^−3^

### 

Data collection: *SMART* (Bruker, 1999 ▶); cell refinement: *SAINT* (Bruker, 1999 ▶); data reduction: *SAINT*; program(s) used to solve structure: *SHELXS97* (Sheldrick, 2008 ▶); program(s) used to refine structure: *SHELXL97* (Sheldrick, 2008 ▶); molecular graphics: *SHELXTL-Plus* (Sheldrick, 2008 ▶); software used to prepare material for publication: *SHELXL97*.

## Supplementary Material

Crystal structure: contains datablocks global, I. DOI: 10.1107/S1600536809043360/vm2007sup1.cif

Structure factors: contains datablocks I. DOI: 10.1107/S1600536809043360/vm2007Isup2.hkl

Additional supplementary materials:  crystallographic information; 3D view; checkCIF report

## Figures and Tables

**Table 1:** Selected geometric parameters (Å, °)

Cd1—N3	2.268 (4)
Cd1—O4	2.268 (4)
Cd1—N1	2.287 (4)
Cd1—O1^i^	2.295 (3)
Cd1—O2^i^	2.504 (3)
Cd1—O3	2.570 (4)

Symmetry code: (i) 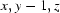.

**Fig. 1.**
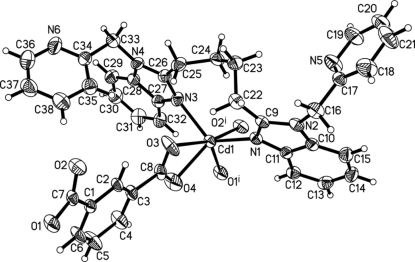
A view of the local coordination of the CdII cations in (I). Displacement ellipsoids are drawn at the 30% probability level. [Symmetry codes: (i) x, -1 + y, z]

**Fig. 2.**
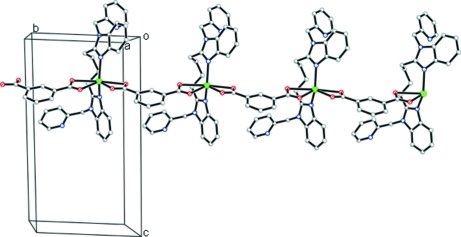
View of the polymeric chain in the title compound. H atoms are omitted for clarity.

## supplementary crystallographic information

### Comment

The design and synthesis of metal-organic coordination polymers are of great interest due to their tremendous potential applications (Zhao *et al.*, 2008). As part of an investigation of these field there is a need to prepare further examples of coordination frameworks. In this paper, the structure of the title compound, (I), is described.

The asymmetric unit of the title compound comprises a cadmium^II^ cation, a btc anion, and a *L* ligand (Fig. 1). The metal centre is coordinated by four O atoms from two btc anions, and two N atoms from one *L* ligand in a distorted octahedral geometry. Each *L* ligand in (I) coordinates to one Cd^II^ cations through its two imidazole N atoms in a bidentate mode. Each btc anion displays bidentate chelating mode, and linked Cd^II^ cations to a chain along *b* axis.

### Experimental

The ligand *L* was synthesized according to the literature but 3-(chloromethyl)pyridine was replaced by 2-(chloromethyl)pyridine (Zhang *et al.*, 2008). A mixture of CdCO_3_ (2 mmol), *L* (2 mmol), and water (8 ml) was sealed in a Teflon reactor (15 ml) and heated at 170 °C for 3 days. After the mixture had been cooled to room temperature at 10 °C.h^-1^, colorless crystals of (I) were obtained.

### Refinement

All H-atoms bound to carbon were refined using a riding model with d(C—H) = 0.93 Å, *U*_iso_=1.2*U*_eq_ (C) for aromatic and 0.97 Å, *U*_iso_ = 1.5*U*_eq_ (C) for CH_2_ atoms.

### Crystal data

**Table:**

[Cd(C_8_H_4_O_4_)(C_30_H_28_N_6_)]	*Z* = 2
*M**_r_* = 749.10	*F*(000) = 764
Triclinic, *P*1	*D*_x_ = 1.515 Mg m^−^^3^
Hall symbol: -P 1	Mo *K*α radiation, λ = 0.71069 Å
*a* = 8.999 (4) Å	Cell parameters from 7395 reflections
*b* = 10.094 (5) Å	θ = 3.1–27.5°
*c* = 19.135 (9) Å	µ = 0.72 mm^−^^1^
α = 91.569 (2)°	*T* = 293 K
β = 97.412 (2)°	Block, colorless
γ = 107.297 (1)°	0.21 × 0.18 × 0.10 mm
*V* = 1641.7 (13) Å^3^	

### Data collection

**Table:**

Bruker APEX CCD area-detector diffractometer	7395 independent reflections
Radiation source: fine-focus sealed tube	5023 reflections with *I* > 2σ(*I*)
graphite	*R*_int_ = 0.054
ω scans	θ_max_ = 27.5°, θ_min_ = 3.1°
Absorption correction: multi-scan (*SADABS*; Sheldrick, 1996)	*h* = −11→11
*T*_min_ = 0.85, *T*_max_ = 0.92	*k* = −13→11
16049 measured reflections	*l* = −24→24

### Refinement

**Table:**

Refinement on *F*^2^	Primary atom site location: structure-invariant direct methods
Least-squares matrix: full	Secondary atom site location: difference Fourier map
*R*[*F*^2^ > 2σ(*F*^2^)] = 0.052	Hydrogen site location: inferred from neighbouring sites
*wR*(*F*^2^) = 0.135	H-atom parameters constrained
*S* = 1.09	*w* = 1/[σ^2^(*F*_o_^2^) + (0.0618*P*)^2^ + 0.1632*P*] where *P* = (*F*_o_^2^ + 2*F*_c_^2^)/3
7395 reflections	(Δ/σ)_max_ = 0.001
442 parameters	Δρ_max_ = 0.62 e Å^−^^3^
0 restraints	Δρ_min_ = −0.73 e Å^−^^3^

### Special details

**Table:**

Geometry. All e.s.d.'s (except the e.s.d. in the dihedral angle between two l.s. planes) are estimated using the full covariance matrix. The cell e.s.d.'s are taken into account individually in the estimation of e.s.d.'s in distances, angles and torsion angles; correlations between e.s.d.'s in cell parameters are only used when they are defined by crystal symmetry. An approximate (isotropic) treatment of cell e.s.d.'s is used for estimating e.s.d.'s involving l.s. planes.
Refinement. Refinement of *F*^2^ against ALL reflections. The weighted *R*-factor *wR* and goodness of fit *S* are based on *F*^2^, conventional *R*-factors *R* are based on *F*, with *F* set to zero for negative *F*^2^. The threshold expression of *F*^2^ > σ(*F*^2^) is used only for calculating *R*-factors(gt) *etc*. and is not relevant to the choice of reflections for refinement. *R*-factors based on *F*^2^ are statistically about twice as large as those based on *F*, and *R*- factors based on ALL data will be even larger.

### Fractional atomic coordinates and isotropic or equivalent isotropic displacement parameters (Å^2^)

**Table:**

	*x*	*y*	*z*	*U*_iso_*/*U*_eq_	
Cd1	1.00480 (4)	0.38577 (3)	0.225104 (18)	0.04123 (13)	
C1	1.1972 (5)	1.0604 (4)	0.2777 (2)	0.0403 (10)	
C2	1.1223 (5)	0.9197 (4)	0.2609 (2)	0.0420 (10)	
H2	1.0213	0.8915	0.2356	0.050*	
C3	1.1944 (5)	0.8208 (4)	0.2807 (3)	0.0466 (11)	
C4	1.3417 (6)	0.8627 (5)	0.3199 (3)	0.0734 (18)	
H4	1.3912	0.7970	0.3338	0.088*	
C5	1.4174 (7)	1.0042 (5)	0.3388 (4)	0.083 (2)	
H5	1.5160	1.0321	0.3661	0.100*	
C6	1.3463 (6)	1.1006 (4)	0.3170 (3)	0.0634 (15)	
H6	1.3982	1.1945	0.3286	0.076*	
C7	1.1201 (5)	1.1677 (4)	0.2559 (2)	0.0424 (10)	
C8	1.1160 (6)	0.6686 (4)	0.2579 (3)	0.0537 (12)	
C9	0.8368 (5)	0.3983 (4)	0.0619 (3)	0.0444 (11)	
C10	0.9382 (5)	0.2779 (4)	−0.0073 (3)	0.0478 (11)	
C11	1.0091 (5)	0.2847 (4)	0.0620 (3)	0.0453 (11)	
C12	1.1305 (5)	0.2252 (5)	0.0782 (3)	0.0528 (12)	
H12	1.1816	0.2307	0.1241	0.063*	
C13	1.1713 (6)	0.1578 (5)	0.0230 (3)	0.0622 (14)	
H13	1.2508	0.1166	0.0323	0.075*	
C14	1.0957 (7)	0.1504 (5)	−0.0465 (3)	0.0674 (15)	
H14	1.1259	0.1042	−0.0822	0.081*	
C15	0.9780 (6)	0.2099 (5)	−0.0627 (3)	0.0618 (13)	
H15	0.9273	0.2050	−0.1087	0.074*	
C16	0.7360 (6)	0.3751 (5)	−0.0689 (3)	0.0583 (13)	
H16A	0.8028	0.4043	−0.1051	0.070*	
H16B	0.6951	0.4508	−0.0578	0.070*	
C17	0.6000 (5)	0.2504 (5)	−0.0985 (3)	0.0477 (11)	
C18	0.5282 (7)	0.2494 (8)	−0.1653 (4)	0.088 (2)	
H18	0.5632	0.3235	−0.1931	0.106*	
C19	0.4334 (7)	0.0358 (6)	−0.0846 (4)	0.087 (2)	
H19	0.4025	−0.0386	−0.0565	0.104*	
C20	0.3551 (7)	0.0276 (7)	−0.1501 (4)	0.081 (2)	
H20	0.2709	−0.0506	−0.1666	0.098*	
C21	0.3998 (9)	0.1337 (9)	−0.1915 (4)	0.108 (3)	
H21	0.3464	0.1303	−0.2368	0.130*	
C22	0.7366 (5)	0.4795 (5)	0.0837 (3)	0.0539 (12)	
H22A	0.7208	0.5396	0.0467	0.065*	
H22B	0.7913	0.5383	0.1258	0.065*	
C23	0.5763 (5)	0.3899 (6)	0.0990 (3)	0.0599 (14)	
H23A	0.5053	0.4460	0.0978	0.072*	
H23B	0.5327	0.3148	0.0625	0.072*	
C24	0.5874 (5)	0.3296 (5)	0.1699 (3)	0.0578 (13)	
H24A	0.6709	0.2866	0.1739	0.069*	
H24B	0.4896	0.2577	0.1730	0.069*	
C25	0.6199 (6)	0.4398 (6)	0.2313 (3)	0.0612 (14)	
H25A	0.6838	0.5279	0.2173	0.073*	
H25B	0.5209	0.4514	0.2404	0.073*	
C26	0.7019 (5)	0.4049 (4)	0.2978 (3)	0.0495 (11)	
C27	0.8663 (5)	0.3384 (4)	0.3716 (3)	0.0503 (11)	
C28	0.7624 (6)	0.3761 (5)	0.4110 (3)	0.0535 (12)	
C29	0.7740 (7)	0.3682 (6)	0.4831 (3)	0.0676 (15)	
H29	0.7049	0.3938	0.5089	0.081*	
C30	0.8936 (8)	0.3203 (6)	0.5157 (3)	0.0782 (17)	
H30	0.9041	0.3127	0.5642	0.094*	
C31	0.9978 (8)	0.2837 (6)	0.4772 (3)	0.0745 (16)	
H31	1.0771	0.2527	0.5005	0.089*	
C32	0.9856 (7)	0.2924 (6)	0.4051 (3)	0.0642 (14)	
H32	1.0558	0.2680	0.3796	0.077*	
C33	0.5476 (6)	0.4866 (5)	0.3825 (3)	0.0650 (14)	
H33A	0.5031	0.4439	0.4229	0.078*	
H33B	0.4624	0.4723	0.3437	0.078*	
C34	0.6237 (6)	0.6427 (5)	0.4007 (3)	0.0575 (13)	
C35	0.7335 (7)	0.7272 (6)	0.3636 (3)	0.0676 (15)	
H35	0.7665	0.6903	0.3255	0.081*	
C36	0.6326 (9)	0.8280 (7)	0.4733 (4)	0.096 (2)	
H36	0.5974	0.8639	0.5110	0.116*	
C37	0.7406 (8)	0.9175 (7)	0.4395 (4)	0.087 (2)	
H37	0.7782	1.0116	0.4540	0.104*	
C38	0.7924 (7)	0.8662 (6)	0.3841 (4)	0.0767 (17)	
H38	0.8671	0.9249	0.3604	0.092*	
N1	0.9426 (4)	0.3586 (4)	0.1048 (2)	0.0430 (9)	
N2	0.8308 (4)	0.3523 (4)	−0.0064 (2)	0.0476 (9)	
N3	0.8258 (5)	0.3584 (4)	0.3010 (2)	0.0519 (10)	
N4	0.6604 (4)	0.4188 (4)	0.3627 (2)	0.0512 (10)	
N5	0.5553 (6)	0.1478 (5)	−0.0578 (3)	0.0749 (14)	
N6	0.5738 (6)	0.6912 (5)	0.4555 (3)	0.0842 (16)	
O1	1.2047 (4)	1.2932 (3)	0.2595 (2)	0.0585 (9)	
O2	0.9777 (4)	1.1328 (3)	0.2335 (2)	0.0655 (10)	
O3	0.9799 (5)	0.6329 (3)	0.2257 (2)	0.0827 (13)	
O4	1.1913 (5)	0.5856 (3)	0.2701 (3)	0.0897 (14)	

### Atomic displacement parameters (Å^2^)

**Table:**

	*U*^11^	*U*^22^	*U*^33^	*U*^12^	*U*^13^	*U*^23^
Cd1	0.04259 (18)	0.02772 (16)	0.0555 (2)	0.01688 (13)	0.00022 (14)	0.00031 (13)
C1	0.043 (2)	0.0257 (18)	0.054 (3)	0.0153 (18)	0.005 (2)	−0.0014 (18)
C2	0.045 (2)	0.0267 (19)	0.050 (3)	0.0090 (18)	0.000 (2)	−0.0058 (18)
C3	0.056 (3)	0.027 (2)	0.057 (3)	0.018 (2)	0.000 (2)	−0.0033 (19)
C4	0.073 (3)	0.037 (2)	0.112 (5)	0.033 (3)	−0.021 (3)	−0.005 (3)
C5	0.063 (3)	0.044 (3)	0.130 (6)	0.022 (3)	−0.042 (3)	−0.013 (3)
C6	0.054 (3)	0.026 (2)	0.101 (4)	0.014 (2)	−0.020 (3)	−0.013 (2)
C7	0.050 (2)	0.029 (2)	0.052 (3)	0.0202 (19)	0.000 (2)	−0.0052 (18)
C8	0.074 (3)	0.026 (2)	0.061 (3)	0.015 (2)	0.010 (3)	−0.001 (2)
C9	0.039 (2)	0.034 (2)	0.053 (3)	0.0036 (19)	−0.002 (2)	0.001 (2)
C10	0.047 (2)	0.038 (2)	0.055 (3)	0.006 (2)	0.007 (2)	0.001 (2)
C11	0.043 (2)	0.035 (2)	0.056 (3)	0.0075 (19)	0.009 (2)	−0.001 (2)
C12	0.047 (3)	0.050 (3)	0.063 (3)	0.017 (2)	0.006 (2)	0.006 (2)
C13	0.055 (3)	0.050 (3)	0.088 (4)	0.021 (2)	0.021 (3)	0.006 (3)
C14	0.077 (4)	0.055 (3)	0.073 (4)	0.016 (3)	0.030 (3)	−0.008 (3)
C15	0.069 (3)	0.055 (3)	0.060 (3)	0.016 (3)	0.016 (3)	0.001 (3)
C16	0.062 (3)	0.051 (3)	0.057 (3)	0.011 (2)	0.002 (2)	0.017 (2)
C17	0.046 (2)	0.048 (3)	0.048 (3)	0.016 (2)	−0.002 (2)	0.000 (2)
C18	0.076 (4)	0.107 (5)	0.075 (5)	0.023 (4)	−0.009 (3)	0.021 (4)
C19	0.078 (4)	0.060 (4)	0.099 (5)	−0.008 (3)	−0.004 (4)	−0.004 (3)
C20	0.058 (3)	0.066 (4)	0.111 (6)	0.017 (3)	−0.012 (4)	−0.026 (4)
C21	0.099 (5)	0.119 (6)	0.088 (5)	0.031 (5)	−0.045 (4)	−0.019 (5)
C22	0.056 (3)	0.043 (2)	0.063 (3)	0.022 (2)	−0.006 (2)	−0.001 (2)
C23	0.047 (3)	0.066 (3)	0.070 (4)	0.029 (2)	−0.004 (2)	−0.005 (3)
C24	0.041 (2)	0.050 (3)	0.080 (4)	0.015 (2)	−0.001 (2)	−0.011 (3)
C25	0.053 (3)	0.063 (3)	0.078 (4)	0.031 (3)	0.014 (3)	0.003 (3)
C26	0.049 (3)	0.038 (2)	0.065 (3)	0.017 (2)	0.012 (2)	0.003 (2)
C27	0.052 (3)	0.036 (2)	0.063 (3)	0.013 (2)	0.007 (2)	0.003 (2)
C28	0.055 (3)	0.042 (2)	0.060 (3)	0.008 (2)	0.016 (2)	0.001 (2)
C29	0.068 (3)	0.070 (4)	0.065 (4)	0.016 (3)	0.021 (3)	0.004 (3)
C30	0.088 (4)	0.082 (4)	0.058 (4)	0.014 (4)	0.014 (3)	0.008 (3)
C31	0.087 (4)	0.070 (4)	0.072 (4)	0.033 (3)	0.007 (3)	0.016 (3)
C32	0.077 (4)	0.062 (3)	0.062 (4)	0.034 (3)	0.011 (3)	0.006 (3)
C33	0.059 (3)	0.057 (3)	0.076 (4)	0.011 (3)	0.015 (3)	−0.007 (3)
C34	0.052 (3)	0.055 (3)	0.069 (4)	0.024 (2)	0.007 (3)	−0.004 (3)
C35	0.074 (3)	0.059 (3)	0.069 (4)	0.019 (3)	0.011 (3)	0.005 (3)
C36	0.102 (5)	0.073 (4)	0.123 (6)	0.038 (4)	0.022 (5)	−0.019 (4)
C37	0.083 (4)	0.057 (3)	0.115 (6)	0.019 (3)	0.000 (4)	−0.008 (4)
C38	0.081 (4)	0.060 (3)	0.080 (5)	0.012 (3)	0.000 (3)	0.006 (3)
N1	0.0398 (18)	0.0382 (18)	0.050 (2)	0.0145 (16)	−0.0029 (16)	−0.0014 (16)
N2	0.044 (2)	0.0358 (18)	0.056 (3)	0.0067 (17)	−0.0027 (18)	0.0052 (17)
N3	0.058 (2)	0.047 (2)	0.059 (3)	0.0276 (19)	0.009 (2)	0.0001 (19)
N4	0.046 (2)	0.045 (2)	0.066 (3)	0.0163 (18)	0.013 (2)	0.0006 (19)
N5	0.076 (3)	0.059 (3)	0.068 (3)	−0.004 (2)	−0.011 (2)	0.010 (2)
N6	0.092 (4)	0.067 (3)	0.103 (4)	0.032 (3)	0.033 (3)	−0.009 (3)
O1	0.0604 (19)	0.0275 (15)	0.087 (3)	0.0199 (15)	−0.0048 (18)	−0.0008 (15)
O2	0.0485 (19)	0.0457 (18)	0.101 (3)	0.0220 (16)	−0.0107 (18)	−0.0053 (18)
O3	0.099 (3)	0.0296 (17)	0.095 (3)	0.0048 (19)	−0.036 (2)	−0.0056 (18)
O4	0.086 (3)	0.0293 (17)	0.155 (4)	0.0280 (19)	0.000 (3)	−0.007 (2)

### Geometric parameters (Å, °)

**Table:**

Cd1—N3	2.268 (4)	C19—N5	1.353 (7)
Cd1—O4	2.268 (4)	C19—H19	0.9300
Cd1—N1	2.287 (4)	C20—C21	1.344 (10)
Cd1—O1^i^	2.295 (3)	C20—H20	0.9300
Cd1—O2^i^	2.504 (3)	C21—H21	0.9300
Cd1—O3	2.570 (4)	C22—C23	1.528 (7)
C1—C2	1.387 (5)	C22—H22A	0.9700
C1—C6	1.389 (6)	C22—H22B	0.9700
C1—C7	1.492 (6)	C23—C24	1.505 (7)
C2—C3	1.380 (6)	C23—H23A	0.9700
C2—H2	0.9300	C23—H23B	0.9700
C3—C4	1.375 (7)	C24—C25	1.533 (7)
C3—C8	1.512 (6)	C24—H24A	0.9700
C4—C5	1.401 (7)	C24—H24B	0.9700
C4—H4	0.9300	C25—C26	1.493 (7)
C5—C6	1.361 (7)	C25—H25A	0.9700
C5—H5	0.9300	C25—H25B	0.9700
C6—H6	0.9300	C26—N3	1.328 (6)
C7—O2	1.237 (5)	C26—N4	1.357 (6)
C7—O1	1.264 (5)	C27—C32	1.384 (7)
C7—Cd1^ii^	2.746 (4)	C27—N3	1.389 (6)
C8—O4	1.234 (6)	C27—C28	1.401 (7)
C8—O3	1.241 (6)	C28—C29	1.376 (7)
C9—N1	1.335 (5)	C28—N4	1.386 (6)
C9—N2	1.364 (6)	C29—C30	1.391 (8)
C9—C22	1.475 (6)	C29—H29	0.9300
C10—C11	1.386 (7)	C30—C31	1.388 (9)
C10—C15	1.388 (7)	C30—H30	0.9300
C10—N2	1.391 (6)	C31—C32	1.376 (8)
C11—N1	1.394 (6)	C31—H31	0.9300
C11—C12	1.401 (6)	C32—H32	0.9300
C12—C13	1.386 (7)	C33—N4	1.463 (6)
C12—H12	0.9300	C33—C34	1.530 (7)
C13—C14	1.403 (8)	C33—H33A	0.9700
C13—H13	0.9300	C33—H33B	0.9700
C14—C15	1.372 (8)	C34—N6	1.330 (7)
C14—H14	0.9300	C34—C35	1.385 (7)
C15—H15	0.9300	C35—C38	1.370 (8)
C16—N2	1.442 (6)	C35—H35	0.9300
C16—C17	1.507 (6)	C36—N6	1.340 (8)
C16—H16A	0.9700	C36—C37	1.360 (10)
C16—H16B	0.9700	C36—H36	0.9300
C17—N5	1.311 (6)	C37—C38	1.361 (9)
C17—C18	1.354 (7)	C37—H37	0.9300
C18—C21	1.403 (9)	C38—H38	0.9300
C18—H18	0.9300	O1—Cd1^ii^	2.295 (3)
C19—C20	1.344 (9)	O2—Cd1^ii^	2.504 (3)
			
N3—Cd1—O4	102.53 (16)	C18—C21—H21	120.5
N3—Cd1—N1	124.31 (14)	C9—C22—C23	113.7 (4)
O4—Cd1—N1	116.42 (15)	C9—C22—H22A	108.8
N3—Cd1—O1^i^	114.31 (14)	C23—C22—H22A	108.8
O4—Cd1—O1^i^	81.94 (13)	C9—C22—H22B	108.8
N1—Cd1—O1^i^	109.42 (13)	C23—C22—H22B	108.8
N3—Cd1—O2^i^	87.25 (13)	H22A—C22—H22B	107.7
O4—Cd1—O2^i^	134.17 (13)	C24—C23—C22	111.9 (4)
N1—Cd1—O2^i^	92.00 (13)	C24—C23—H23A	109.2
O1^i^—Cd1—O2^i^	53.91 (10)	C22—C23—H23A	109.2
N3—Cd1—O3	82.04 (15)	C24—C23—H23B	109.2
O4—Cd1—O3	52.87 (13)	C22—C23—H23B	109.2
N1—Cd1—O3	90.93 (13)	H23A—C23—H23B	107.9
O1^i^—Cd1—O3	134.73 (12)	C23—C24—C25	112.3 (4)
O2^i^—Cd1—O3	168.63 (14)	C23—C24—H24A	109.1
C2—C1—C6	118.6 (4)	C25—C24—H24A	109.1
C2—C1—C7	121.6 (4)	C23—C24—H24B	109.1
C6—C1—C7	119.9 (4)	C25—C24—H24B	109.1
C3—C2—C1	121.4 (4)	H24A—C24—H24B	107.9
C3—C2—H2	119.3	C26—C25—C24	113.7 (4)
C1—C2—H2	119.3	C26—C25—H25A	108.8
C4—C3—C2	119.1 (4)	C24—C25—H25A	108.8
C4—C3—C8	120.0 (4)	C26—C25—H25B	108.8
C2—C3—C8	120.9 (4)	C24—C25—H25B	108.8
C3—C4—C5	120.2 (4)	H25A—C25—H25B	107.7
C3—C4—H4	119.9	N3—C26—N4	111.7 (4)
C5—C4—H4	119.9	N3—C26—C25	124.1 (5)
C6—C5—C4	119.9 (5)	N4—C26—C25	124.2 (4)
C6—C5—H5	120.0	C32—C27—N3	131.3 (5)
C4—C5—H5	120.0	C32—C27—C28	120.0 (5)
C5—C6—C1	120.8 (4)	N3—C27—C28	108.6 (4)
C5—C6—H6	119.6	C29—C28—N4	132.2 (5)
C1—C6—H6	119.6	C29—C28—C27	122.0 (5)
O2—C7—O1	121.6 (4)	N4—C28—C27	105.8 (4)
O2—C7—C1	120.1 (4)	C28—C29—C30	117.0 (5)
O1—C7—C1	118.2 (4)	C28—C29—H29	121.5
O2—C7—Cd1^ii^	65.6 (2)	C30—C29—H29	121.5
O1—C7—Cd1^ii^	56.1 (2)	C29—C30—C31	121.4 (6)
C1—C7—Cd1^ii^	173.7 (3)	C29—C30—H30	119.3
O4—C8—O3	122.6 (4)	C31—C30—H30	119.3
O4—C8—C3	118.8 (5)	C32—C31—C30	121.1 (6)
O3—C8—C3	118.5 (4)	C32—C31—H31	119.4
O4—C8—Cd1	54.3 (2)	C30—C31—H31	119.4
O3—C8—Cd1	68.3 (2)	C31—C32—C27	118.4 (6)
C3—C8—Cd1	173.1 (4)	C31—C32—H32	120.8
N1—C9—N2	111.0 (4)	C27—C32—H32	120.8
N1—C9—C22	125.8 (4)	N4—C33—C34	112.0 (4)
N2—C9—C22	123.2 (4)	N4—C33—H33A	109.2
C11—C10—C15	123.1 (5)	C34—C33—H33A	109.2
C11—C10—N2	105.9 (4)	N4—C33—H33B	109.2
C15—C10—N2	131.0 (5)	C34—C33—H33B	109.2
C10—C11—N1	109.1 (4)	H33A—C33—H33B	107.9
C10—C11—C12	119.9 (4)	N6—C34—C35	122.5 (5)
N1—C11—C12	131.0 (5)	N6—C34—C33	113.7 (5)
C13—C12—C11	117.3 (5)	C35—C34—C33	123.8 (5)
C13—C12—H12	121.4	C38—C35—C34	118.8 (6)
C11—C12—H12	121.4	C38—C35—H35	120.6
C12—C13—C14	121.7 (5)	C34—C35—H35	120.6
C12—C13—H13	119.2	N6—C36—C37	124.0 (7)
C14—C13—H13	119.2	N6—C36—H36	118.0
C15—C14—C13	121.2 (5)	C37—C36—H36	118.0
C15—C14—H14	119.4	C36—C37—C38	118.6 (6)
C13—C14—H14	119.4	C36—C37—H37	120.7
C14—C15—C10	116.9 (5)	C38—C37—H37	120.7
C14—C15—H15	121.6	C37—C38—C35	119.3 (6)
C10—C15—H15	121.6	C37—C38—H38	120.4
N2—C16—C17	114.7 (4)	C35—C38—H38	120.4
N2—C16—H16A	108.6	C9—N1—C11	106.3 (4)
C17—C16—H16A	108.6	C9—N1—Cd1	130.4 (3)
N2—C16—H16B	108.6	C11—N1—Cd1	123.2 (3)
C17—C16—H16B	108.6	C9—N2—C10	107.6 (4)
H16A—C16—H16B	107.6	C9—N2—C16	128.7 (4)
N5—C17—C18	122.9 (5)	C10—N2—C16	123.7 (4)
N5—C17—C16	118.2 (4)	C26—N3—C27	106.4 (4)
C18—C17—C16	119.0 (5)	C26—N3—Cd1	128.9 (3)
C17—C18—C21	118.1 (6)	C27—N3—Cd1	120.4 (3)
C17—C18—H18	120.9	C26—N4—C28	107.5 (4)
C21—C18—H18	120.9	C26—N4—C33	127.9 (4)
C20—C19—N5	122.6 (6)	C28—N4—C33	123.9 (5)
C20—C19—H19	118.7	C17—N5—C19	118.1 (5)
N5—C19—H19	118.7	C34—N6—C36	116.9 (6)
C21—C20—C19	119.2 (6)	C7—O1—Cd1^ii^	96.7 (3)
C21—C20—H20	120.4	C7—O2—Cd1^ii^	87.6 (2)
C19—C20—H20	120.4	C8—O3—Cd1	85.0 (3)
C20—C21—C18	119.1 (6)	C8—O4—Cd1	99.4 (3)
C20—C21—H21	120.5		
			
C6—C1—C2—C3	−1.7 (7)	O4—Cd1—N1—C9	−88.1 (4)
C7—C1—C2—C3	179.5 (4)	O1^i^—Cd1—N1—C9	−178.6 (3)
C1—C2—C3—C4	2.0 (8)	O2^i^—Cd1—N1—C9	129.1 (4)
C1—C2—C3—C8	−176.0 (4)	O3—Cd1—N1—C9	−39.9 (4)
C2—C3—C4—C5	−0.5 (9)	C7^i^—Cd1—N1—C9	153.8 (4)
C8—C3—C4—C5	177.5 (6)	C8—Cd1—N1—C9	−62.4 (4)
C3—C4—C5—C6	−1.4 (11)	N3—Cd1—N1—C11	−134.7 (3)
C4—C5—C6—C1	1.8 (11)	O4—Cd1—N1—C11	96.0 (3)
C2—C1—C6—C5	−0.3 (9)	O1^i^—Cd1—N1—C11	5.5 (3)
C7—C1—C6—C5	178.6 (6)	O2^i^—Cd1—N1—C11	−46.7 (3)
C2—C1—C7—O2	13.2 (7)	O3—Cd1—N1—C11	144.3 (3)
C6—C1—C7—O2	−165.7 (5)	C7^i^—Cd1—N1—C11	−22.0 (3)
C2—C1—C7—O1	−165.3 (4)	C8—Cd1—N1—C11	121.8 (3)
C6—C1—C7—O1	15.9 (7)	N1—C9—N2—C10	0.5 (5)
C4—C3—C8—O4	−5.8 (8)	C22—C9—N2—C10	−178.9 (4)
C2—C3—C8—O4	172.2 (5)	N1—C9—N2—C16	−178.4 (4)
C4—C3—C8—O3	176.4 (6)	C22—C9—N2—C16	2.3 (7)
C2—C3—C8—O3	−5.7 (8)	C11—C10—N2—C9	−1.3 (4)
N3—Cd1—C8—O4	115.1 (4)	C15—C10—N2—C9	−180.0 (5)
N1—Cd1—C8—O4	−118.4 (4)	C11—C10—N2—C16	177.6 (4)
O1^i^—Cd1—C8—O4	−1.3 (4)	C15—C10—N2—C16	−1.1 (7)
O2^i^—Cd1—C8—O4	27.8 (6)	C17—C16—N2—C9	−106.1 (5)
O3—Cd1—C8—O4	−177.0 (6)	C17—C16—N2—C10	75.3 (6)
C7^i^—Cd1—C8—O4	8.1 (5)	N4—C26—N3—C27	1.0 (5)
N3—Cd1—C8—O3	−68.0 (3)	C25—C26—N3—C27	−179.9 (4)
O4—Cd1—C8—O3	177.0 (6)	N4—C26—N3—Cd1	−155.2 (3)
N1—Cd1—C8—O3	58.6 (4)	C25—C26—N3—Cd1	23.9 (7)
O1^i^—Cd1—C8—O3	175.7 (3)	C32—C27—N3—C26	−179.6 (5)
O2^i^—Cd1—C8—O3	−155.2 (4)	C28—C27—N3—C26	−0.5 (5)
C7^i^—Cd1—C8—O3	−174.9 (3)	C32—C27—N3—Cd1	−21.0 (7)
C15—C10—C11—N1	−179.5 (4)	C28—C27—N3—Cd1	158.1 (3)
N2—C10—C11—N1	1.7 (5)	O4—Cd1—N3—C26	90.1 (4)
C15—C10—C11—C12	2.2 (7)	N1—Cd1—N3—C26	−44.7 (4)
N2—C10—C11—C12	−176.6 (4)	O1^i^—Cd1—N3—C26	176.8 (4)
C10—C11—C12—C13	−1.7 (6)	O2^i^—Cd1—N3—C26	−135.2 (4)
N1—C11—C12—C13	−179.6 (4)	O3—Cd1—N3—C26	41.0 (4)
C11—C12—C13—C14	0.6 (7)	C7^i^—Cd1—N3—C26	−158.4 (4)
C12—C13—C14—C15	0.1 (8)	C8—Cd1—N3—C26	65.8 (4)
C13—C14—C15—C10	0.2 (8)	O4—Cd1—N3—C27	−63.3 (3)
C11—C10—C15—C14	−1.4 (7)	N1—Cd1—N3—C27	162.0 (3)
N2—C10—C15—C14	177.1 (4)	O1^i^—Cd1—N3—C27	23.5 (4)
N2—C16—C17—N5	16.9 (7)	O2^i^—Cd1—N3—C27	71.5 (3)
N2—C16—C17—C18	−163.9 (5)	O3—Cd1—N3—C27	−112.3 (3)
N5—C17—C18—C21	0.5 (10)	C7^i^—Cd1—N3—C27	48.2 (3)
C16—C17—C18—C21	−178.7 (6)	C8—Cd1—N3—C27	−87.5 (3)
N5—C19—C20—C21	0.8 (11)	N3—C26—N4—C28	−1.1 (5)
C19—C20—C21—C18	0.7 (12)	C25—C26—N4—C28	179.8 (4)
C17—C18—C21—C20	−1.3 (11)	N3—C26—N4—C33	169.6 (4)
N1—C9—C22—C23	−92.5 (5)	C25—C26—N4—C33	−9.5 (7)
N2—C9—C22—C23	86.7 (6)	C29—C28—N4—C26	−179.9 (5)
C9—C22—C23—C24	77.3 (5)	C27—C28—N4—C26	0.7 (5)
C22—C23—C24—C25	71.7 (5)	C29—C28—N4—C33	8.9 (8)
C23—C24—C25—C26	−153.7 (4)	C27—C28—N4—C33	−170.5 (4)
C24—C25—C26—N3	46.1 (7)	C34—C33—N4—C26	−86.9 (6)
C24—C25—C26—N4	−135.0 (5)	C34—C33—N4—C28	82.4 (6)
C32—C27—C28—C29	−0.4 (7)	C18—C17—N5—C19	0.9 (9)
N3—C27—C28—C29	−179.6 (4)	C16—C17—N5—C19	−179.9 (5)
C32—C27—C28—N4	179.1 (4)	C20—C19—N5—C17	−1.6 (10)
N3—C27—C28—N4	−0.1 (5)	C35—C34—N6—C36	1.2 (9)
N4—C28—C29—C30	−179.6 (5)	C33—C34—N6—C36	−178.3 (6)
C27—C28—C29—C30	−0.3 (8)	C37—C36—N6—C34	−1.2 (11)
C28—C29—C30—C31	0.7 (9)	O2—C7—O1—Cd1^ii^	4.7 (5)
C29—C30—C31—C32	−0.5 (9)	C1—C7—O1—Cd1^ii^	−176.9 (4)
C30—C31—C32—C27	−0.2 (9)	O1—C7—O2—Cd1^ii^	−4.3 (5)
N3—C27—C32—C31	179.6 (5)	C1—C7—O2—Cd1^ii^	177.3 (4)
C28—C27—C32—C31	0.6 (8)	O4—C8—O3—Cd1	2.9 (6)
N4—C33—C34—N6	−140.2 (5)	C3—C8—O3—Cd1	−179.3 (4)
N4—C33—C34—C35	40.3 (8)	N3—Cd1—O3—C8	110.7 (3)
N6—C34—C35—C38	−0.2 (9)	O4—Cd1—O3—C8	−1.7 (3)
C33—C34—C35—C38	179.2 (5)	N1—Cd1—O3—C8	−124.8 (3)
N6—C36—C37—C38	0.3 (12)	O1^i^—Cd1—O3—C8	−5.8 (4)
C36—C37—C38—C35	0.7 (10)	O2^i^—Cd1—O3—C8	130.3 (6)
C34—C35—C38—C37	−0.7 (9)	C7^i^—Cd1—O3—C8	11.1 (6)
N2—C9—N1—C11	0.6 (5)	O3—C8—O4—Cd1	−3.3 (6)
C22—C9—N1—C11	179.9 (4)	C3—C8—O4—Cd1	178.9 (4)
N2—C9—N1—Cd1	−175.8 (3)	N3—Cd1—O4—C8	−68.0 (4)
C22—C9—N1—Cd1	3.5 (6)	N1—Cd1—O4—C8	71.0 (4)
C10—C11—N1—C9	−1.5 (5)	O1^i^—Cd1—O4—C8	178.7 (4)
C12—C11—N1—C9	176.6 (4)	O2^i^—Cd1—O4—C8	−166.5 (3)
C10—C11—N1—Cd1	175.3 (3)	O3—Cd1—O4—C8	1.7 (3)
C12—C11—N1—Cd1	−6.7 (6)	C7^i^—Cd1—O4—C8	−173.9 (4)
N3—Cd1—N1—C9	41.1 (4)		

Symmetry codes: (i) *x*, *y*−1, *z*; (ii) *x*, *y*+1, *z*.
